# Misdiagnosis of brachial plexus schwannoma as cervical radiculopathy

**Published:** 2013

**Authors:** Mahnaz Khajepour, Alireza Ghazizadeh Ehsayei, Hossein Salehi, Mansour Raygani, Darioush Eliaspour

**Affiliations:** 1 Physiatrist, University of Social Welfare and Rehabilitation Sciences, Tehran, Iran; 2 Department of Hematology and Oncology AND Shariati Hospital, Tehran University of Medical Sciences, Tehran, Iran; 3 Assistant Professor, Department of surgery AND Aliebneabitaleb Hospital, Rafsanjan University of Medical Sciences, Rafsanjan, Iran; 4 Professor, Department of Physical Medicine and Rehabilitation AND Shohada-Tajrish Hospital, Shahid Beheshti University of Medical Sciences, Tehran, Iran; 5 Assistant Professor, Department of Physical Medicine and Rehabilitation AND Shohada-Tajrish Hospital, Shahid Beheshti University of Medical Sciences, Tehran, Iran

**Keywords:** Brachial Plexus, Schwannoma, Radiculopathy

## Case Report

A 51-year-old woman referred to Ali-Ibn-Abitaleb hospital (Rafsanjan, Iran) with chronic cervical pain, radiating to left forearm since about 5 years ago. Pain was vague and the patient had no sensory complaint at first, but there had been recent exacerbation of pain, since 6 months ago, as sharp radiating pain and paresthesia in 4^th^ and 5^th^ digits of left hand.

**Figure 1:**
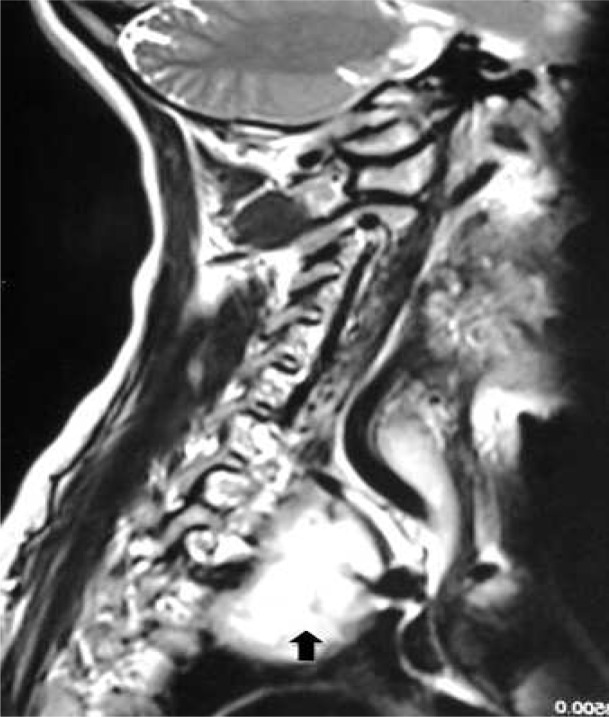
Mass lesion at left root of neck, unspecified origin (arrow)

The first clinical suspicion of physicians was cervical radiculopathy but in magnetic resonance imaging (MRI) there was no disc protrusion/extrusion in cervical and upper thoracic region, and there was a mass lesion at left root of neck (Figure 1).

Despite this result the patient was treated as cervical radiculopathy with collars, NSAIDs, and gabapentin for 3 months.

With the persistence of pain and paresthesia, she came to our clinic. On the first physical examination, cervical bending was limited by pain, Spurling's maneuver was negative, and pain was aggravated by arm abduction and flexion. There was also mild atrophy of the first interdigital space. By electrodiagnostic examination, increased latency and low amplitude was detected in the left medial antebrachial cutaneous nerve (about 35% of right side). Moreover, in needle electromyography there was increased insertional activity, spontaneous discharges and neurogenic motor units in first dorsal interosseous, extensor indicis, flexor carpi radialis, and left abductor pollicis brevis muscle. The brachioradialis muscle was normal. These findings were compatible with mixed brachial plexus lesion, severe in lower trunk and mild to moderate in medial cord (Table 1). In sonography of the cervical region, there was a soft tissue mass in posterior triangle with the size of 63 × 46 mm with sharp borders, and it was approved by MRI (Figure 1).

**Table 1:** Electrodiagnostic findings of the patient

Nerve		Latency (ms)	Amplitude (uv)	Nerve conduction velocity (uv)
Medial antebrachial cutaneous-Left		2.3	5.57	-
Medial antebrachial cutaneous-Right		1.7	16.0	-
Ulnar (sensory)-Left		3.1	35.0	-
Median (sensory)-Right		3.2	39.5	-
Ulnar (motor)-Left	distal	2.8	4.7	58.9
	proximal	5.5	4.4	
Median (motor)-Right	distal	3.2	5.0	59.0
	proximal	5.7	4.5	

The patient was consulted for surgical excision; open excision through anterior approach (supraclavicular) was done. The prominent bulk of tumor was located in superior mediastinum with extension to neck and serious adhesion to lower part of left brachial plexus especially lower trunk.

Pathological assessment revealed totally encapsulated neoplasm composed of spindle cells with no mitosis or necrosis. The final diagnosis was schwannoma. After tumor excision the patient had significant and immediate relief of pain and paresthesia but there was some residual numbness in medial aspect of left forearm and hand after 2 months due to neuropraxia.

Schwannomas, neurilemomas, or neurinomas are relatively rare, but benign nerve sheath tumors derive from Schwann cells with low tendency of transformation to malignancy. They are the most common neurogenic tumors and arise from any nerve surrounded by Schwann cells such as cranial, autonomic, or peripheral nerves.

Solitary schwannomas of the head and neck are uncommon tumors arising from any cranial or autonomic nerve.^1–4^ 25% to 45% of extracranial schwannomas occur in the head and neck. A total of 28 consecutive patients were treated between January 2000 and August 2006, for solitary schwannomas in different major nerves of the head and neck. Most affected trunks were cranial nerves in 14 patients (50%), cervical sympathetic chain in 7 (25%), and brachial plexus in 7 (25%). The most common sign was an isolated well-demarcated lesion placement at the lateral aspect of the neck for those tumors arising from vagus, lingual, and sympathetic nerves.^2^

Extracranial schwannomas usually present insidiously, and thus are often diagnosed incorrectly or after lengthy delays. The most common physical findings associated with brachial plexus tumors are related to local growth. Motor or sensory deficits caused by axonal loss occur more frequently with neurofibromas and malignant nerve sheath tumors. Schwannomas are usually seen as an asymptomatic neck mass, so difficulties in diagnosis are common.^3–5^

Furthermore, the cervical region is a limited space for many vascular and neural structures (such as nerve trunks, plexus, and the sympathetic chain); so any mass in this region, accompanied by sensory or motor symptoms or signs, can be a serious alarm for more investigations. In this case the chief complaint of the patient was pain and numbness in upper limb. We could not find any mass in physical examination of the neck, because the main bulk of tumor was in upper mediastinum with extension to brachial plexus.

## Conclusion

In case of radiating pain and paresthesia in upper limb (as this case) symptoms can be misleading and may suggest cervical radiculopathy. However, careful examination, especially in case of persistence of symptoms and negative imaging results for radiculopathies, is important.
